# Plant-Produced Glycosylated and In Vivo Deglycosylated Receptor Binding Domain Proteins of SARS-CoV-2 Induce Potent Neutralizing Responses in Mice

**DOI:** 10.3390/v13081595

**Published:** 2021-08-12

**Authors:** Tarlan Mamedov, Damla Yuksel, Merve Ilgın, Irem Gurbuzaslan, Burcu Gulec, Hazel Yetiskin, Muhammet Ali Uygut, Shaikh Terkis Islam Pavel, Aykut Ozdarendeli, Gulshan Mammadova, Deniz Say, Gulnara Hasanova

**Affiliations:** 1 Department of Agricultural Biotechnology, Akdeniz University, 07058 Antalya, Turkey; dmlyuksel07@gmail.com (D.Y.); merveilgin.akd@gmail.com (M.I.); irem.gurbuzaslan@gmail.com (I.G.); burcudogusoy@gmail.com (B.G.); gulka2878@gmail.com (G.M.); denizsy13@gmail.com (D.S.); gulnarahasanova@yahoo.com (G.H.); 2 Department of Microbiology, Medical Faculty, Erciyes University, 38280 Kayseri, Turkey; hazelyetiskin@gmail.com (H.Y.); mauygut@gmail.com (M.A.U.); biotech.pavel@outlook.com (S.T.I.P.); aozdarendeli@erciyes.edu.tr (A.O.); 3 Vaccine Research, Development and Application Center, Erciyes University, 38280 Kayseri, Turkey

**Keywords:** COVID-19, SARS-CoV-2, spike protein, receptor binding domain (RBD) vaccine, plant, transient expression system

## Abstract

The COVID-19 pandemic, caused by SARS-CoV-2, has rapidly spread to more than 222 countries and has put global public health at high risk. The world urgently needs cost-effective and safe SARS-CoV-2 vaccines, antiviral, and therapeutic drugs to control it. In this study, we engineered the receptor binding domain (RBD) of the SARS-CoV-2 spike (S) protein and produced it in the plant *Nicotiana benthamiana* in a glycosylated and deglycosylated form. Expression levels of both glycosylated (gRBD) and deglycosylated (dRBD) RBD were greater than 45 mg/kg fresh weight. The purification yields were 22 mg of pure protein/kg of plant biomass for gRBD and 20 mg for dRBD, which would be sufficient for commercialization of these vaccine candidates. The purified plant-produced RBD protein was recognized by an S protein-specific monoclonal antibody, demonstrating specific reactivity of the antibody to the plant-produced RBD proteins. The SARS-CoV-2 RBD showed specific binding to angiotensin converting enzyme 2 (ACE2), the SARS-CoV-2 receptor. In mice, the plant-produced RBD antigens elicited high titers of antibodies with a potent virus-neutralizing activity. To our knowledge, this is the first report demonstrating that mice immunized with plant-produced deglycosylated RBD form elicited high titer of RBD-specific antibodies with potent neutralizing activity against SARS-CoV-2 infection. Thus, obtained data support that plant-produced glycosylated and in vivo deglycosylated RBD antigens, developed in this study, are promising vaccine candidates for the prevention of COVID-19.

## 1. Introduction

The novel coronavirus, currently designated as SARS-CoV-2, is a highly pathogenic coronavirus, spread to more than to 222 countries and territories in a short time, more than 199,022,838 cases have been recorded, with more than 4,240,374 confirmed deaths (https://www.worldometers.info/coronavirus/ accessed on 2 August 2021). Although various types of vaccines (mRNA, adenovirus vector vaccines, and inactivated vaccines) are now available and some other ones (mRNA, DNA, and viral vector-based, protein-based subunits, inactivated, attenuated) are underway, the world still urgently needs more safe and effective, alternative SARS-CoV-2 vaccines, antiviral and therapeutic drugs, cost-effective diagnostic reagents and kits to control the COVID-19 pandemic and relieve the human suffering associated with the pandemic that kills thousands of people every day.

One of the major observed features of this virus is that SARS-CoV-2 is transmitted from infected people without symptoms; therefore, it increases the challenges of controlling a deadly pandemic without the use of a vaccine. One-third of the virus genome (~30 kb) of SARS-CoV-2 encodes mainly structural proteins, such as the spike (S) glycoprotein, nucleocapsid protein (N), small envelop protein (E) and matrix protein (M). When reported in January 2020, in spike protein, SARS-CoV-2 had about ~80% sequence identity with that of SARS-CoV [1]. Now the sequence identity is less than 75% as the spike protein of SARS-CoV-2 has acquired more than 725 mutations [2]. SARS-CoV-2 virus undergoes about two mutations per month in the global population [3,4]. The D614G strain of SARS-CoV-2 virus demonstrated an increased sensitivity to neutralizing antibodies, probably resulting from the mutation of the S protein’s molecular dynamics. [5,6,7]. Crystal structures of the S protein of SARS-CoV-2 as well as the complex of RBD (receptor binding domain) with ACE2 (angiotensin-converting enzyme 2) revealed that the RBD contains a core and a receptor-binding motif (RBM), which forms contact with ACE2 [8]. The virus infectivity, human-to-human transmission as well as immune escape are impacted by RBM mutations. [9]. The N501Y mutation (within the RBD region) was found in B.1.1.7 (Alpha, British variant). In addition, the E484K mutation, which is also within the RBD region, has been recently reported to occur in different variants and has been found in the South African (Beta, B.1.351) and Brazilian (B.1.1.28, with mutations similar to B.1.351) variants. It was shown that the N439K mutation, which is also within the RBD region, confers resistance against several neutralizing monoclonal antibodies (mAbs) [10]. Sensitivity of a few neutralizing mAbs to mutations at positions 417 (K417V) and 439 (N439K) has also been reported in other studies [11,12,13,14]. A new variant, Delta (B.1.617) has been detected in many countries. Compared with the D614G strain, the S protein of Delta contains nine mutations, including five mutations in the NTD, two mutations in the RBD (L452R, T478K), one mutation close to the furin cleavage site (P681R), and one in the S2 region (D950N) [15]. It should be noted that mutations within the RBD observed for Y453, G476, F486, T500, and N501 are close to the ACE-2 receptor binding site [16]. Mutations affecting the interaction between the S protein and the ACE-2 receptor could potentially affect the effectiveness of the vaccines and also drugs, which were designed to operate at the protein–protein interface.

The S protein of coronavirus consists of S1 and S2 domains and plays a key role in virus binding, fusion, and entry into host cells. Notably, the intrinsic mechanical stability of the SARS-CoV-2 S–ACE2 interaction has been shown to be larger than SARS-CoV and is key during the structural transition from close to open states and key for infection at the early stage [17]. Thus, identification of the potential target sites to destabilize the spike protein could be used to discover new drugs that can inhibit spike protein conformations that enable binding to ACE2. S protein has been demonstrated as a leading target for vaccine and neutralizing antibody development. In fact, most of vaccines against COVID-19, such as mRNA, DNA, viral vector-based, subunits, and protein-based, have been developed based on the gene encoding the S protein [18,19,20,21,22]. S1 domain contains an RBD, which binds specifically to ACE2, the receptor for both SARS-CoV and SARS-CoV-2. It was shown that the RBD alternates between two distinct conformational states relative to the remainder of the spike: “open” and “closed” [23,24,25]. A conserved allosteric pocket adjacent to a hinge region was recently identified as critical for the RBD opening and closing motion [26]. The S protein of SARS-CoV-2 is a cysteine-rich protein, and nine cysteine residues are found in the RBD, eight of which are involved in forming four pairs of disulfide bridges [22]. Thus, the selection of amino acid regions of RBDs is critical for correct disulfide bridge formation, hence for proper folding of the produced recombinant SARS-CoV-2 RBD. In addition, the S protein has 22 potential N-glycosylation sites, and two of them are in the RBD [27]. Recent studies reported a structural role of N-glycans at N165 and N234 as modulators of RBD conformational dynamics [28,29]. Thus, the correct formation of disulfide bridges and status of glycosylation are essential for functional activity of recombinant S protein-based vaccines, produced using different expression systems. The structure of SARS-CoV-2 spike RBD was recently determined by crystal structure analysis at 2.45 Å resolution [23,30]. It was demonstrated that the overall ACE2-binding mode of the SARS-CoV-2 RBD is nearly identical to that of the SARS-CoV RBD, which also utilizes ACE2 as the cell receptor. It should be noted that since the RBD is the critical region for receptor binding, RBD-based vaccines could be promising for developing vaccines and highly potent cross-reactive therapeutic agents. It has been recently demonstrated that an RBD-Fc-based COVID-19 vaccine candidate elicited high titer of RBD-specific antibodies with neutralizing activity against SARS-CoV-2 infections [31].

Numerous studies in recent years have demonstrated that a plant expression system is a promising expression platform for the cost-effective, rapid, and safe production of various recombinant proteins. A plant expression system has several advantages over other expression systems currently in use. It is generally employed for producing pharmaceutical products, such as vaccine candidates, therapeutic proteins, enzymes, and antibodies [15,16,17,18]. Recently, several flexible approaches (co-expression with deglycosylation enzymes to achieve in vivo furin processing of target proteins, co-expression with molecular chaperons, protein engineering, glycan engineering, engineering the plant secretory pathway) have been developed, which enabled the successful production of pharmaceutically important complex proteins, including human proteins and enzymes, in plants [32,33,34,35,36,37,38,39,40,41]. Thus, a plant expression system could be the ideal platform for a cost-effective, safe, and rapid production of structural proteins of SARS-CoV-2 as a vaccine candidate against COVID-19. In previous efforts, a number of studies have been conducted on transgenic (stable) expression of the spike protein of SARS-CoV in plants. N-terminal fragment of SARS-CoV S protein (S1) was produced in tomato and low-nicotine tobacco plants and demonstrated immunogenicity in mice after parenteral or oral administration [42]. Several groups have reported development of S protein recombinant plant-based vaccines against different coronaviruses for oral delivery that elicit protective immunity against a virus challenge [43,44,45]. Recombinant SARS-CoV spike protein was also expressed in plant cytosol and chloroplasts [46]. However, the transgenic plant approach has some concerns, which are mainly associated with the long development time. In addition, low target protein accumulations in transgenic plants, as well as the possibility of gene transfer from transgenic plants to wild types, are a serious concern in using the transgenic plant approach [47]. The plant transient expression system has a number of advantages over stable expression. The production of glycosylated forms of RBD SARS-CoV-2 a transient expression system in the plant *Nicotiana benthamiana* has been reported recently [31,48]. However, the expression levels of RBD in *N. benthamiana* reported in these studies were unsatisfactory—8 to 25 μg/g, which is too low to be economical for commercialization. In this study, we report a high-level production of functionally active RBD antigens (glycosylated and deglycosylated forms) using a transient expression platform in *N. benthamiana*. We demonstrated that plant-produced gRBD and dRBD proteins of SARS-CoV-2 showed specific binding to angiotensin-converting enzyme 2 (ACE2), the SARS-CoV-2 receptor. Furthermore, in mice, plant-produced RBD antigens elicited high titers of antibodies with potent virus neutralizing activity.

## 2. Materials and Methods

### 2.1. Cloning and Expression RBD Proteins in N. benthamiana

The gene encoding RBD of SARS-CoV-2 spike protein (receptor-binding domain containing fragment, RBD, 319-591AA, GenBank accession MN985325), FLAG- or His6-tagged, was codon optimized for expression in *N. benthamiana* and de-novo synthesized at Biomatik Corp. (Kitchener, ON, Canada). To transiently express RBD in *N. benthamiana*, the *Nicotiana tabacum* PR-1a signal peptide (MGFVLFSQLPSFLLVSTLLLFLVISHSCRA) was added to the N-terminus of RBD. In addition, the KDEL sequence, the endoplasmic reticulum (ER) retention signal and the FLAG epitope were added to the C-terminus. The resulting sequences were inserted into the pEAQ [49] binary expression vectors to obtain pEAQ-RBD. These plasmids were then transferred into *Agrobacterium* AGL1 strain. To express the dRBD variant in *N. benthamiana*, AGL1 harboring pEAQ-RBD plasmid was infiltrated into *N. benthamiana* plant leaves. Plants were harvested at 4 and 5 dpi (days post infiltration). To produce dRBD, the AGL1 strain harboring pEAQ-RBD was co-infiltrated with pGreenII–Endo H construct [36].

### 2.2. Expression Screening of RBD Proteins Produced in N. benthamiana Plant by Western Blot Analysis

SDS-PAGE analysis of plant-produced gRBD and dRBD proteins was performed on 12% acrylamide gels stained with Coomassie Blue (Gel Code Blue, Pierce Rockford, IL, USA). Western blot analysis was performed after electrophoresis and transfer of the proteins to polyvinylidene fluoride membranes. After transfer, Western blot membranes were blocked with I-Block (Applied Biosystems, Carlsbad, CA, USA), and recombinant proteins were detected with an anti-FLAG antibody, anti-SARS-CoV-2 S protein monoclonal antibody (cat. no. 945102, BioLegend, San Diego, CA, USA), or anti-RBD polyclonal antibody (MBS2563840, MyBioSource, San Diego, CA, USA). The image was taken using highly sensitive GeneGnome XRQ Chemiluminescence imaging system (Syngene, a division of Synoptics Ltd., Cambridge, UK).

### 2.3. Purification of Plant-Produced gRBD and dRBD Proteins Using Anti-DYKDDDDK Affinity Gel

Purification of plant-produced gRBD and dRBD proteins were performed by anti-FLAG affinity chromatography using anti-DYKDDDDK affinity gel (cat. no. 651503, BioLegend) as described previously [37]. For purification, 20 g of frozen leaves, infiltrated with the pEAQ-RBD-Flag-KDEL (with or without pGreenII-Endo H) constructs were ground in 20 mL PBS buffer (1 × PBS, 150 mM NaCl) using a mortar and a pestle. Plant debris was removed by filtration through Miracloth followed by centrifugation at 20,000 g for 25 min, and the supernatant was filtered through a 0.45 μm syringe filter (Millipore, Darmstadt, Germany). An anti-FLAG affinity column was prepared according to the manufacturer’s instructions. Sixty milliliters of a clear supernatant were loaded onto 0.5 mL resin column equilibrated with PBS buffer. The column was washed with 10 volumes of PBS buffer. Bound proteins were eluted using 200 mM glycine, 150 mM NaCl, pH 2.2, into tubes containing 2.0 M Tris solution to neutralize the acidic glycine. Eluted proteins were buffer exchanged against 1 × PBS buffer, concentrated with a Millipore 10K MWCO Amicon Ultra 4 concentrator (cat. no: UFC8010, Millipore), and the total protein content was estimated using the BioDrop and then analyzed by SDS-PAGE and Western blot. The purification yield of purified proteins was calculated and quantified based on SDS-PAGE and WB analysis using highly sensitive Gene Tools software (Syngene Bioimaging, Cambridge, UK) and ImageJ software as described previously [38].

### 2.4. Gel Filtration

Gel filtration was performed with ÄKTA start on a 60 cm × 16 mm column (cat. no. 19-5003-01, GE Healthcare, Chicago, IL, USA), packed with Sephacryl^®^ S-200 HR (cat. no. 17-0584-10, GE Healthcare). The column was equilibrated with 50 mM phosphate buffer, 150 mM NaCl, pH 7.4., and 0.25 mg plant-produced dRBD and gRBD proteins, purified using FLAG affinity chromatography, were loaded onto a column. Eluted fractions were combined and concentrated, and buffer exchanged against PBS and concentrated with a Millipore 10K MWCO Amicon Ultra 4 concentrator (cat. no.: UFC8010, Millipore).

### 2.5. Glycoprotein Detection 

The presence of glycans in plant-produced, purified gRBD and dRBD proteins (glycosylated and in vivo deglycosylated by Endo H) was detected by Pro-Q Emerald 300 glycoprotein staining. About 250 ng of the plant-produced RBD and protective antigen (PA83) variants (glycosylated and in vivo deglycosylated form) were separated on a 10% SDS-PAGE gel, and then the glycans were detected in the gel using the Pro-Q Emerald Glycoprotein Stain Kit according to the manufacturer’s protocol (Pro-Q1Emerald 300 Glycoprotein Gel Stain Kit, with SYPRO1Ruby Protein Gel Stain, P21855, Life technologies, Carlsbad, CA, USA).

### 2.6. Binding Affinity of the Plant-Produced RBD Variants to ACE2

Briefly, 96-well plates (Greiner Bio-One GmbH, Frickenhausen, Germany) were coated with 100 ng of plant-produced gRBD, dRBD, commercially available recombinant RBD protein of the SARS-CoV-2 S protein (cat. no. 793606, BioLegend, USA), or COVID 19 spike protein RBD (active protein, sequence positions Arg319-Phe541, cat. no. MBS2563882, MyBiosource, San Diego, CA, USA) using 100 mM carbonate buffer and incubated overnight at 4 °C. After incubation, the plates were blocked with blocking buffer for 2 h at room temperature. After blocking, various concentrations (50–1000 ng) of commercially available recombinant human ACE2 (cat. no. 792004, BioLegend, USA) were added and incubated for 2 h at 37 °C. After 2 h, purified anti-human ACE2 mAb was added into each well. The plate was washed three times with blocking solution (200 μL/well). After washing, wells were incubated with anti-rat IgG antibody (cat. no. MBS440123, MyBioSource, San Diego USA). The plate was washed three times with 1 × PBST washing solution (200 μL/well) for 5 min. 200 μL of Substrate Solution (Sigma, Ronkonkoma, NY, USA) was added to each well. Afterwards, the plate was incubated in the dark, for 30 min at room temperature. After the incubation period, the plate was read at 450 nm on a multi-well plate reader.

### 2.7. Stability Assessment of Plant-Produced RBD Variants

The stability of plant-produced gRBD or dRBD recombinant proteins of SARS-CoV-2 was examined after incubation at 37 °C for 24, 48, 96, 72, and 96 h using similar procedure as described previously [37]. Briefly, protein samples were diluted to 0.5 mg/mL with PBS and were transferred into polypropylene Eppendorf low-binding tubes. After incubation at 37 °C for 24, 48, 96, 72, and 96 h, samples were mixed with 5 × SDS loading dye and stored at −20 °C until used. For the stability assessment, samples were analyzed by SDS-PAGE and Western blotting (WB). The degradation of gRBD and dRBD protein bands was calculated and quantified based on SDS-PAGE and WB analysis using the highly sensitive Gene Tools software (Syngene Bioimaging, Cambridge, UK) and ImageJ software as described previously [37].

### 2.8. Immunogenicity Studies of gRBD and dRBD Proteins in Mice

Groups of seven-week-old mice (Balb/c male, 6 animals/group) were immunized IM with 5 μg of gRBD or dRBD adsorbed to 0.3% Alhydrogel (Brenntag Biosector, Frederikssund, Denmark) at 0 and 21 days. IgG titers were determined by ELISA in sera collected on day 21 or 42 post vaccination from mice immunized with gRBD or dRBD variants. Flat-bottom 96-well ELISA plates were coated with commercial spike protein or plant-produced gRBD or dRBD proteins (100 ng per well) diluted with coating buffer to detect antibody levels in the sera of mice vaccinated with gRBD or dRBD proteins. We used 10^2^ to 10^8^ dilutions of the sera for ELISA immunogenicity analyses. Endpoint titer referred to reciprocal serum dilutions that gave a mean OD value four times greater than the pre-immune control samples. Control group received PBS with Alhydrogel. Mice studies were carried out at Akdeniz University Experimental Animal Care Unit under permission of the Local Ethics Committee for Animal Experiments at Akdeniz University with the supervision of a veterinarian (Protocol number: 1155/2020.07.01). The animal protocols were approved by the Local Ethics Committee for Animal Experiments at Akdeniz University. All animal experiments and methods were performed in accordance with the animal experimentation guidelines and regulations approved by the Local Ethics Committee for Animal Experiments at Akdeniz University. The study was carried out in compliance with the ARRIVE guidelines.

### 2.9. Virus Titration

Vero E6 cells (African green monkey kidney) obtained from ATCC (CRL 1586) were maintained in Dulbecco’s modified Eagle’s medium (DMEM) supplemented with 10% heat-inactivated fetal bovine serum (FBS) and 100 mM L-glutamine (Sigma–Aldrich, Darmstadt, Germany). The hCoV-19/Turkey/ERAGEM-001/2020 strain was used for titration assay [50]. The SARS-CoV-2 titer was determined by the tissue culture infective dose 50% (TCID50) method. Briefly, Vero E6 cells (0.4 × 10^6^ cells/mL) were seeded in 96-well plates and incubated for 18–24 h at 37 °C. Serial 10-fold dilutions of virus-containing samples were added to 96-well culture plate and cultured for 5–7 days in 5% CO_2_ incubator at 37 °C, and cells were observed for cytopathic effect (CPE) under a microscope. The TCID_50_ was determined according to the Reed and Muench method. All experiments with infectious SARS-CoV-2 were performed in biosafety level 3 (BSL3) enhanced facility at Erciyes University Vaccine Research, Development and Application Center (ERAGEM).

### 2.10. Micro Neutralization Test (MNT)

SARS-CoV-2--specific neutralizing antibody was determined using a microneutralization test (MNT). Serum samples were heat-inactivated at 56 °C for 1 h prior to use. Sera dilutions were 1/8–1/1024 in 2-fold increments and were incubated with the SARS-CoV-2 (100 TCID50) for 1 h at 37 °C. The mixtures were then transferred to 96-well plates containing confluent Vero E6 cell monolayers. The inocula were decanted, and DMEM with 2% FBS was added. The microplates were formatted to test three wells for each serum dilution, six virus-only control wells, and three blank wells containing growth medium alone. Cells were assessed daily for cytopathic effect and listed at 3–4 days post infection (dpi). The 50% neutralization titer (NT50) was calculated as the highest dilution of the serum at which the infectivity was neutralized in 50% of the cell in wells. Seropositivity was defined as a titer ≥1/8.

### 2.11. Statistical Analysis

GraphPad Prism software was used for all statistical analyses. One-way ANOVA test was used for all experiments. It was used to study the binding affinity of gRBD, dRBD, or commercial RBDs of SARS-CoV-2 to ACE2. One-way ANOVA test was also used to compare antibody responses of sera immunized by gRBD or dRBD and to test the neutralization ability of the sera from mice immunized with the plant-produced gRBD and dRBD proteins against live SARS-CoV-2 infection. Results were considered statistically significant at *p* < 0.05 and *p* values were showed as * *p* < 0.05; ** *p* < 0.01; *** *p* < 0.001. Each point on the graphs was derived from three replicas for each dilution.

## 3. Results

### 3.1. Engineering, Cloning, Expression, Purification, and Characterization of RBD Proteins from N. benthamiana 

**Figure 1:**
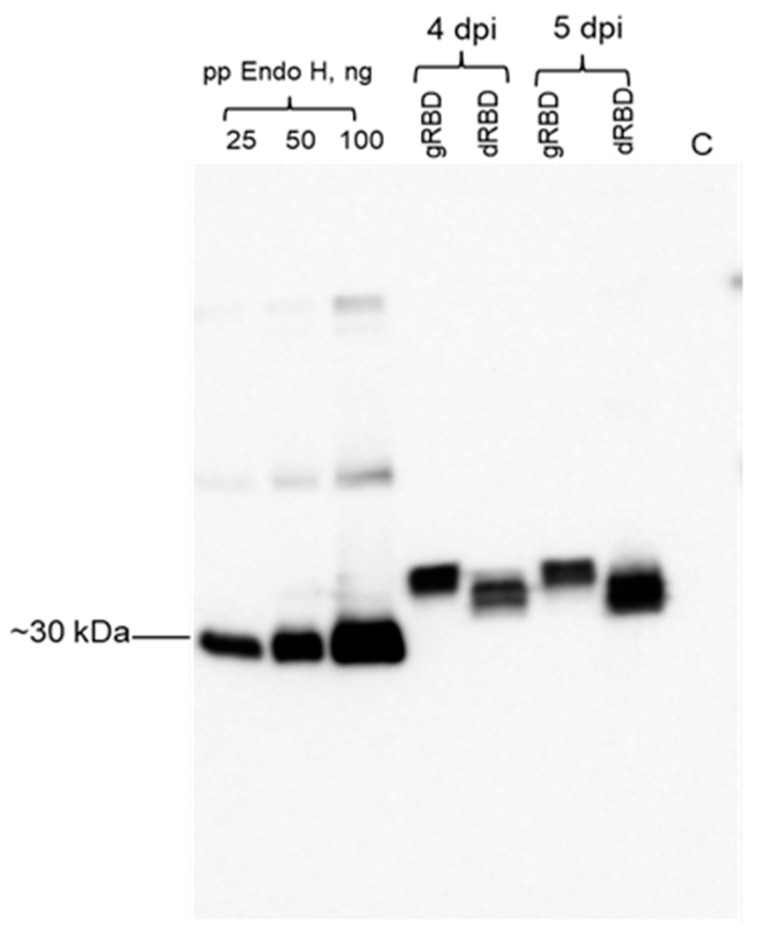
Western blot analysis of the expression of RBD (receptor binding domain) and co-expression of RBD with bacterial Endo H in *N. benthamiana*. *N. benthamiana* plants were infiltrated with pEAQ-RBD or with pEAQ-RBD/pGreenII-Endo H (non-tagged) constructs, to produce glycosylated RBD (gRBD) or in vivo deglycosylated RBD (dRBD) of SARS-CoV-2, respectively. Leaf samples were collected at 4 dpi and 5 dpi (days post-infiltration) and were homogenized in three volumes of extraction buffer. RBD protein bands were probed using the anti-4×His tag mAb. Purified plant-produced FLAG-tagged Endo H protein (~30 kDa, 25, 50, and 100 ng) was used as a standard protein. C—crude extract prepared from a control, non-infiltrated plant. gRBD—glycosylated RBD; dRBD—deglycosylated RBD; pp-plant produced.

In this study, we produced both a glycosylated and non-glycosylated variant of SARS-CoV-2 RBD in the plant *N. benthamiana* (Figure 1). Engineering, codon optimization, cloning, and expression of RBD (R319–S591) were performed as described in Materials and Methods. The deglycosylated form of RBD was produced by co-expression of RBD gene of SARS-CoV-2 with bacterial deglycosylase Endo H [36]. The expression levels determined by Western blot analysis and ELISA were 42 mg/kg plant leaf for deglycosylated and 45 mg/kg for glycosylated forms of RBD. Glycosylated and deglycosylated plant-produced RBD proteins were purified from crude extracts by single-step anti-FLAG antibody affinity chromatography. The purification yields were 22 and 20 mg pure protein/kg plant biomass for gRBD and dRBD, respectively.

**Figure 2:**
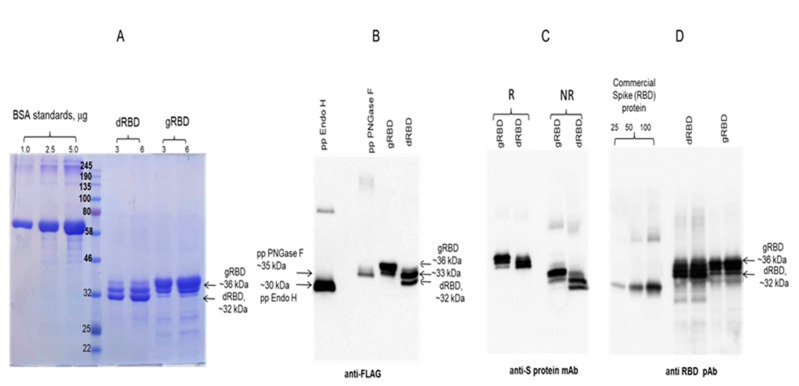
SDS-PAGE analysis of purified plant-produced gRBD and dRBD proteins. RBD protein variants were purified from *N. benthamiana* using anti-FLAG antibody resin. (**A**) gRBD—3 or 6 μg plant-produced glycosylated RBD; dRBD—3 or 6 μg plant-produced deglycosylated RBD; BSA standards—1.0, 2.5 and 5.0 μg; M—color prestained protein standard (NEB). (**B**) Western blot analysis of gRBD and dRBD using anti-Flag antibody: pp Endo H—plant-produced and purified Endo H protein with molecular mass of ~30 kDa [37]. pp PNGase F—plant-produced and purified PNGase F protein with a molecular mass of ~35 kDa [36]. (**C**) Western blot analysis of gRBD and dRBD using commercially available, purified anti-SARS-CoV-2 S protein S1 mAb (cat. no. 945102, BioLegend). (**D**) Western blot analysis of gRBD and dRBD using commercially available anti-RBD antibody (cat. no. MBS2563840, MyBiosource, San Diego, CA, USA); commercial spike protein—COVID 19 spike protein (RBD) active protein, sequence positions Arg319–Phe541 (MBS2563882, MyBiosource); pp—plant-produced; R—reducing; NR—non-reducing condition.

SDS-PAGE and Western blot analysis of the anti-FLAG column purified deglycosylated and glycosylated variants of RBD are shown in Figure 2. The purity of both proteins was higher than 90%. Plant-produced, purified glycosylated RBD molecules appeared as a double protein with a molecular mass (MM) of ~33 or 36 kDa (Figure 2, gRBD). dRBD molecules also appeared as a double protein with a MM of ~32 or ~33 kDa (Figure 2, dRBD). The ~33 kDa band observed in the purified gRBD sample might be due to the presence of a deglycosylated form, observed when RBD was co-expressed with bacterial Endo H.

**Figure 3:**
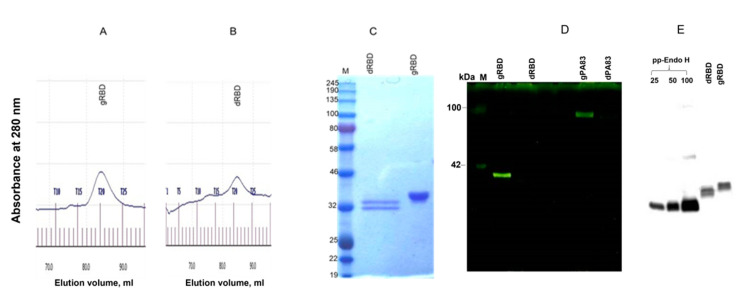
Gel filtration chromatography and glycan detection of plant-produced gRBD or dRBD proteins. Profiles of plant-produced gRBD (**A**) and dRBD (**B**) proteins, eluted from Sephacryl^®^ S-200 HR column. The column was equilibrated with 50 mM phosphate buffer (with 150 mM NaCl, pH 7.4). The plant-produced dRBD and gRBD proteins (0.25 mg), purified using FLAG affinity chromatography, were loaded onto column. Gel filtration was performed with ÄKTA start using 60 cm × 16 mm column (cat. no. 19-5003-01, GEHealthcare, Chicago, IL, USA), packed with Sephacryl^®^ S-200 HR (cat. no. 17-0584-10, GE Healthcare). (**C**) SDS-PAGE analysis of plant-produced gRBD and dRBD proteins eluted from Sephacryl^®^ S-200 HR column. (**D**) Glycan detection in gRBD and dRBD proteins. Protein (250 ng) from each sample (eluted from Sephacryl^®^ S-200 HR column) was separated on a 10% SDS-PAGE gel followed by in-gel glycan detection using the Pro-Q Emerald 300 glycoprotein staining kit. Stained proteins were visualized by UV illumination. M—CandyCane glycoprotein molecular weight standards (Molecular Probes), 250 ng of each protein per lane; gRBD—glycosylated RBD; dRBD—deglycosylated RBD; gPA83—glycosylated PA83; dPA83—deglycosylated PA83. (**E**) Western blot analysis of the same sample using the anti-FLAG antibody. Purified Endo H protein (25, 50, and 100 ng) and gRBD or dRBD proteins (50 ng) were loaded into wells.

Plant-produced dRBD and gRBD proteins were very well recognized by anti-FLAG (Figure 2B) and by S protein-specific monoclonal (Figure 2C) or polyclonal antibodies (Figure 2D), demonstrating specific reactivity of SARS-CoV-2 specific mAb or pAb to the plant-produced gRBD and dRBD proteins. Interestingly, as can be seen in Figure 2D, the RBD produced by the plant differed significantly from the commercial RBD in apparent molecular weight; the plant-produced RBD (aa 319–591) was 50 amino acids longer than the commercial RBD (aa 319–541). Both gRBD and Endo H in vivo deglycosylated RBD (dRBD) eluted as a single peak from the Sephacryl S-200 column and were present as monomers and no dimerization or aggregation were observed (Figure 3A,B). Based on the SDS-PAGE analysis (Figure 3C), the purity of gRBD or dRBD proteins after gel filtration was higher than 95%. Sephacryl S-200 column-purified gRBD and dRBD protein were subjected to glycan detection to demonstrate the in vivo deglycosylation of target proteins by Endo H, as described in Materials and Methods. As shown in Figure 3D, the glycan was only detected in plant-produced glycosylated RBD and glycosylated PA83. Figure 3E shows the Western blot analysis of the same samples (diluted 1:5) probed with an anti-FLAG antibody. These results are similar to those of our earlier determination of glycans in the PA83 and Pfs48/45–10C proteins in vivo deglycosylated by Endo H [37] or the Pfs48/45 protein in vivo deglycosylated by PNGase F [33].

### 3.2. Binding of Plant-Produced RBD Variants to ACE2

**Figure 4:**
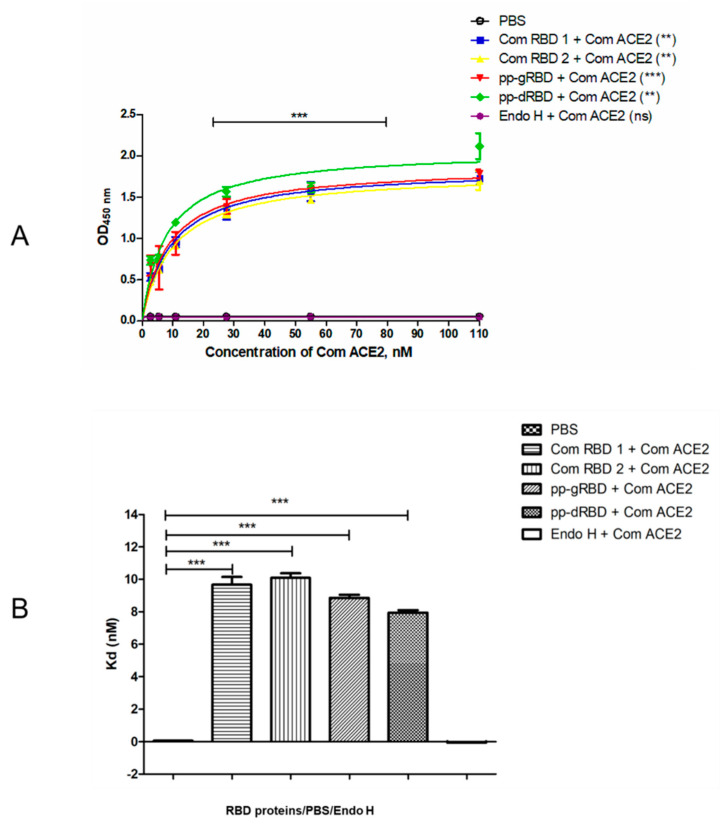
Binding affinity of gRBD, dRBD, and commercial RBDs of SARS-CoV-2 to ACE2, the receptor of SARS-CoV-2. The samples of 100 ng of plant-produced gRBD, dRBD, and commercially available (**A**) recombinant SARS-CoV-2 S protein RBD (amino acids Arg319–Phe541, with a C-terminal 8×His tag, expressed in 293E cells, cat. no. 793606, BioLegend, USA), or (**B**) SARS-CoV-2 spike protein (RBD) coronavirus active protein (amino acids Arg319–Phe541, produced in baculovirus–insect cells, cat. no. MBS2563882, MyBioSource, San Diego, CA, USA) were incubated on plates coated with ACE2. After incubation, anti-SARS-CoV-2 spike RBD polyclonal antibodies were added into each well and detected with rabbit IgG conjugated with HRP. Each point on the graph was derived from three replica for each dilution. Data are shown as mean ± standard error of the mean (SEM) of triplicates in each sample dilution. Statistical significance (*p* < 0.05) was calculated using the one-way ANOVA test with Tukey’s multiple comparisons. *p* value for each group is shown in parentheses. ** *p* < 0.01, *** *p* < 0.001; OD—optical density; Kd—dissociation constant.

Binding of plant-produced gRBD and dRBD proteins, along with commercial RBDs, to ACE2 was assessed as described in Materials and Methods. As can be seen from Figure 4, the plant-produced RBD variants demonstrated specific binding to ACE2, the SARS-CoV-2 receptor. Notably, Endo H in vivo deglycosylated RBD exhibited a slightly stronger binding (Kd = 7.88 ± 0.062 nM) to ACE2 compared with its glycosylated (Kd = 8.816 ± 0.054 nM) counterpart.

### 3.3. Stability Assessment of RBD Proteins

**Figure 5:**
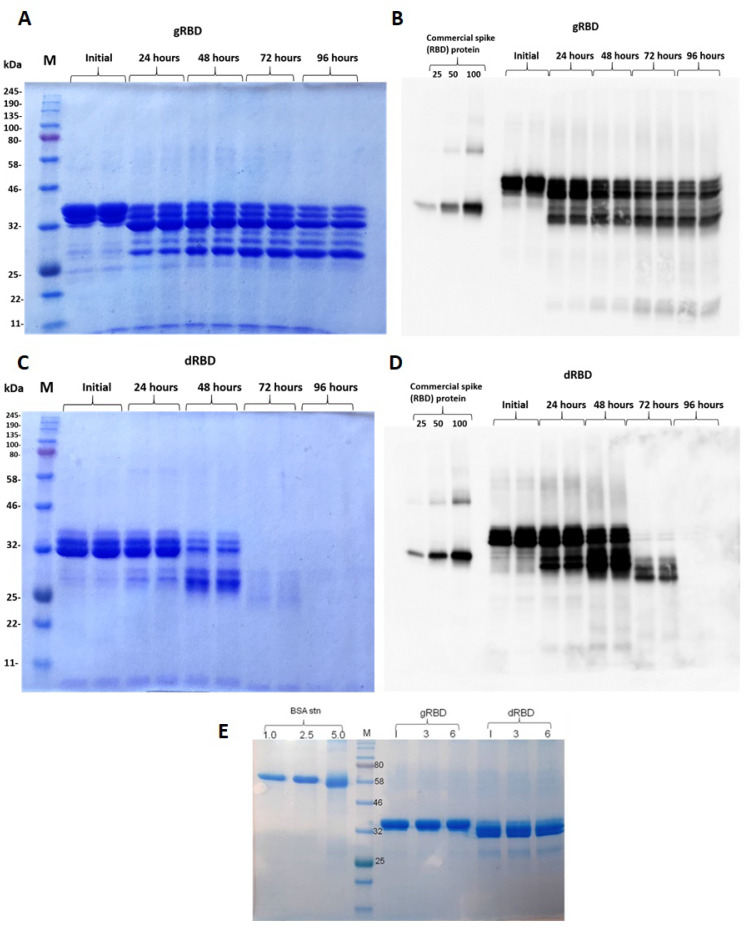
Stability of glycosylated and deglycosylated RBD proteins. Plant-produced, FLAG-antibody affinity column-purified glycosylated (gRBD, **A**,**B**) or in vivo Endo H deglycosylated (dRBD, **C**,**D**) proteins were incubated at 37 °C for 24, 48, 72, and 96 h and analyzed in SDS-PAGE (**A**,**C**) and Western blotting (**B**,**D**). Lanes were loaded with ~4.0 μg of gRBD (**A**) or dRBD (**C**) and ~200 ng of gRBD (**B**) or dRBD (**D**). M—color prestained protein standard. Proteins on the blot were probed with anti-SARS-CoV-2 spike RBD polyclonal antibodies. (**E**) Proteins were stored at −80 °C for 3 and 6 months as indicated and then analyzed using SDS-PAGE: I—initial sample, after purification of gRBD or dRBD proteins, SDS sample buffer was added to it and it was stored at −80 °C. The image was taken using the highly sensitive GeneGnomeXRQ Chemiluminescence imaging system. Protein bands were calculated and quantified based on SDS-PAGE and WB analyses using the highly sensitive Gene Tools software (Syngene Bioimaging, Cambridge, UK) and ImageJ software, as described previously [38].

The stability of gRBD and dRBD of SARS-CoV was assessed by SDS-PAGE analysis after incubation at 37 °C for 24, 48, 72, and 96 h. The procedure is described in Materials and Methods. These analyses showed that dRBD degradation was very low during incubation at 37 °C for 24 h (less than 10%); however, under the same conditions, gRBD degraded significantly (more than 60%) (Figure 5). There was almost no degradation of plant-produced gRBD or dRBD proteins when stored at −80 °C for 6 months (Figure 5E).

### 3.4. Immunogenicity Studies of gRBD and dRBD Variants in Mice

**Figure 6:**
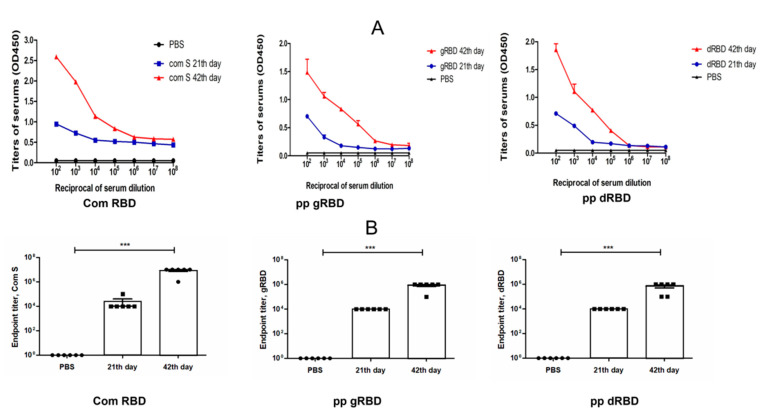
Immunogenicity of plant-produced RBD variants in mice. Mice (6–7-week-old Balb/c male) were immunized on days 0 and 21 IM with 5 μg (with alum) of plant-produced gRBD and dRBD using Alhydrogel as an adjuvant. IgG titers were determined by ELISA in sera collected on days 21 or 42 post-vaccination from mice immunized with plant-produced gRBD and dRBD. For immunogenicity analysis, 10^2^ to 10^8^ dilutions of sera were assessed by ELISA. Endpoint titer refers to reciprocal serum dilutions that give a mean OD value four times greater than the pre-immune control samples. Control group received PBS with Alhydrogel. (**A**) Detection of the SARS-CoV-2 RBD-specific IgG in different dilutions of sera from day 21 or 42. (**B**) Detection of IgG titers in the murine serum with different dilutions specific to commercially available S protein, gRBD, dRBD at day 21 or 42. Each point on the graph was derived from three replicas for each dilution. Data are shown as mean ± standard error of the mean (SEM) of triplicates in each sample dilution. Statistical significance (*p* < 0.05) was calculated using the one-way ANOVA test with Tukey’s multiple comparisons. *** *p* < 0.001 (n = 6 mice/group).

Mice received two doses of each variant, gRBD or dRBD proteins, adsorbed to 0.3% Alhydrogel at three-week intervals (0, 21 days). Serum samples were collected on days −1 (pre-bleed), 21 (post first vaccination), and 42 (post second vaccination) and assessed for anti-gRBD or anti-dRBD antibody responses with an IgG ELISA. IgG responses showed that plant-produced RBD protein variants were able to induce significantly high titers of antibodies with alum adjuvant at the 5 μg dose after 21 days and more after 47 days (Figure 6).

### 3.5. Neutralization Activity Assessment of gRBD and dRBD by Micro Neutralization Test

**Figure 7:**
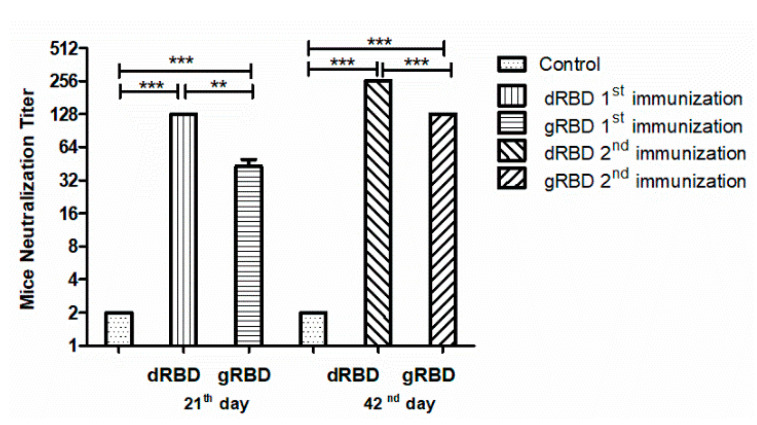
In vitro microneutralization assay of mouse sera against live SARS-CoV-2 in the Vero E6 cell line immunized with plant-produced gRBD or dRBD. In the assay, 32 to 1024 dilutions of the sera were used. Statistical significance (*p* < 0.05) was calculated using the one-way ANOVA test. ** *p* < 0.01; *** *p* < 0.001; n = 6 mice/group.

We examined the neutralization ability of sera from mice immunized with the plant-produced gRBD and dRBD proteins against live SARS-CoV-2 infection in Vero-E6 cells. The neutralization titers for gRBD and dRBD are presented in Figure 7. Sera collected from mice immunized with 5 μg plant-produced dRBD (adjuvanted with Alhydrogel), collected at day 21 post-first immunization, had a three-fold greater neutralization titer compared with plant-produced gRBD. Murine sera collected from mice immunized with 5 μg plant-produced gRBD or dRBD proteins (adjuvanted with Alhydrogel), collected at day 42 post-second immunization, displayed SARS-CoV-2 neutralization activity at 1:128 or 1:256 dilutions, respectively. Thus, both plant-produced SARS-CoV-2 RBD variants induced potent neutralizing responses in mice and sera immunized with the plant-produced SARS-CoV-2 dRBD variant had a greater neutralizing activity against live SARS-CoV-2 compared with its glycosylated counterpart, gRBD.

## 4. Discussion

SARS-CoV-2 is a novel and highly pathogenic coronavirus, which has caused an outbreak in Wuhan City, China, in 2019 and then spread rapidly throughout the world. The new coronavirus disease, COVID-19, is currently responsible for the pandemic creating a huge global human health crisis with significant negative impacts on health and economies worldwide. Although several COVID-19 vaccines are currently available, approved for human use, it is very important to develop cost-effective, safe vaccines and therapeutic proteins to prevent the spread of the disease and protect populations.

The genomes of all known coronaviruses, including SARS-CoV, MERS-CoV, and SARS-CoV-2, encode four structural proteins: N, S, E, and M. A number of studies [41,51,52,53,54,55,56,57] have demonstrated that the S protein plays a key role in the receptor (ACE2) recognition and is responsible for attachment to the host receptor. It has been demonstrated that the binding affinity between ACE2 and the RBD of SARS-CoV-2 is much stronger than that of SARS-CoV [58], which logically explains the increased infectivity of SARS-CoV-2 versus SARS-CoV. The RBD domain of the S protein has been considered as a potential and leading target for vaccine development against SARS-CoV-2. Particularly, the RBD has been shown to contain multiple conformation-dependent epitopes and, therefore, induces immune responses and highly potent neutralizing antibodies [28,29,59,60]. The S protein of SARS-CoV-2 and its receptor, the ACE2 protein, are heavily glycosylated, and several N-glycosylation sites are close to the binding sites [61,62,63,64]. The S-glycoprotein of SARS-CoV-2 has 22 potential N-glycosylation sites [27], while the human ACE2 has 7 [63,64,65]. Thus, the virus (SARS-Cov-2) and receptor (ACE2) binding affinity on the surface of human cells could be a critical step in the viral entry into the susceptible cells. Notably, numerous studies have demonstrated that protein-based subunit vaccines have many advantages over other vaccine types, particularly recombinant protein-based subunit vaccines are largely considered to be safer [66,67,68].

A plant transient expression system is a promising expression platform for the production of a variety of recombinant proteins, such as vaccines, antibodies, therapeutic proteins, and enzymes. Using a transient expression system, a number of difficult-to-express proteins have been successfully produced in *N. benthamiana*, such as the full length Pfs48/45 of *Plasmodium falciparum* [36,38], human factor IX and furin, and the heptamerized form of PA63 of *Bacillus anthracis* [38]. The major challenge in any expression system, including plant expression systems, is to achieve the production of recombinant proteins with native folding, native-like post-translational modifications (PTM), high solubility, and high yield. Flexible approaches are required for successful production of functionally active recombinant proteins in plants with high yields. In this study, we achieved the production of functionally active glycosylated and in vivo deglycosylated variants of RBD (consisting of amino acids R319–S591) of SARS-CoV-2 structural protein at a high level and in a highly soluble form in the plant *N. benthamiana* using a transient expression platform [69]. Production of the glycosylated form of RBD (aa F318–C617), alone [48] or fused with immunoglobulin Fc domain [31], using a transient plant expression system in *N. benthamiana*, has been recently reported. The expression levels of these RBD proteins produced in *N. benthamiana* were low (8 to 25 μg/g). As mentioned above, the S protein of SARS-CoV-2 is cysteine-rich and nine cysteine residues are found in the RBD, eight of which are involved in forming four pairs of disulfide bridges [61]. One of the reasons why low expression levels were observed for the RBD, covering amino acids F318–C617 [31], could be due to the remaining unpaired cysteine at position 617 [48], which forms a disulfide bridge with C649 in the full-length S protein [24]. It should be noted that the production of target proteins with an Fc domain can be a useful tool for the expression of some proteins; however, it is very important to produce the protein as a native protein as it is required for proper folding, stability, and functional activity. In other words, although the circulating half-life of some drugs can be improved by expressing them as fusion proteins (for instance, with Fc [70] or albumin [70]) the fusion adds extra amino acids to the sequence of the target protein and, therefore, significantly negatively affects protein folding, product yield, and activity. At this point, the expression level of ACE2-Fc fusion was reported as 100 mg/kg plant leaf [71] versus ~700 mg/kg plant leaf of non-fusion ACE2, produced in *N. benthamiana* [72]. Importantly, non-fused plant-produced ACE2 showed strong anti-SARS-CoV-2 activity (IC50 of 1.02 mg/mL) [72] compared with ACE2-Fc (IC50 of 94.66 mg/mL) [71] at the pre-entry stage of SARS-CoV-2 infection. An unsatisfactory expression level (2–4 μg/g fresh weight) was also observed for His-tagged RBD variant (aa R319–F541), reported by Diego-Martin et al. [73] and Shin et al. [29], which was too low to be economical for commercialization. RBD (aa 319–533, where the number of cysteine residues is even, so they can stabilize the protein conformation) has been produced as a secreted soluble protein with a complex N-glycan structure [29] in a glycoengineered *N. benthamiana* plant. The authors reported a purification yield of RBD (amino acids 319–533), which was comparable to our yield (~10–20 mg/g fresh weight) reported earlier in BioRxiv [69]. The RBD variant that we produced in this study consisted of amino acids R319–S591, so there was an even number of cysteine residues in the amino acid sequence. These data confirm that the correct selection of the amino acid region of the RBD is crucial for high-yield production of a functionally active and soluble protein.

For the expression of RBD in plants, our approach was to engineer the RBD sequence by fusing the C-terminus with the ER retention signal (KDEL) to produce RBD in ER, which is equipped with molecular chaperones and folding assistants, thus enables protein folding as well as post-translational modifications of target proteins [40,74]. The other advantage of the targeting to ER is that ER enables the expression of target glycoproteins with high mannose N-glycan structure that is common in humans, yeast, and plants. Notably, in earlier studies, the carbohydrate epitopes of plant complex glycoproteins have been shown to generate an allergic response in humans [75].

The S protein of SARS-CoV-2 is a cysteine-rich protein, and nine cysteine residues are found in the RBD, eight of which form four pairs of disulfide bridges [61]. In addition, the S protein has 22 potential N-glycosylation sites, two of which (N331 and N343) are in the RBD [27]. It has been shown that two N-glycosylation sites are fully N-glycosylated when expressed in a eukaryotic expression system [64,76]. Based on mutagenesis of the N-glycosylating sites, it has been shown that N-glycosylation is critical to produce functional recombinant RBD variants in plants [29]. It is noteworthy that through amino acid substitution, mutations of N-glycosylated sites can significantly affect folding and properties of the protein [18]. In this study, to understand the role of N-glycosylation, we produced both glycosylated and non-glycosylated variants of the RBD protein in *N. benthamiana*. A deglycosylated RBD variant was produced by co-expression of the SARS-CoV-2 RBD gene with bacterial Endo H—the in vivo deglycosylation strategy that we recently developed [36]. Endo H deglycosylation strategy has been successfully used for production of non-glycosylated forms of functionally active complex proteins, such as full length Pfs48/45 of *Plasmodium falciparum* [37], monomeric and heptameric forms of PA83 of *Bacillus anthracis* [32,35,36,37], and human ACE2 enzyme [58]. We purified this plant-produced gRBD and dRBD proteins using a single step anti-FLAG affinity chromatography. The purification yields were ~22 and ~20 mg/kg plant leaf for glycosylated and deglycosylated forms, respectively. We constructed two plasmids, expressing FLAG-tagged and His-tagged variants of RBD. We found that the expression level and purification yield of His-tagged RBD (Figure S1) was low (less than 10 mg/kg leaf biomass); however, the expression level and purification yield of plant-produced FLAG-tagged RBD protein was significantly higher, more than 20 mg/kg leaf biomass.

The purified plant-produced RBD proteins were recognized by S protein-specific monoclonal and polyclonal antibodies, demonstrating specific reactivity of mAb or pAb to plant-produced RBD variants. The plant-produced glycosylated and deglycosylated RBD proteins showed specific binding to ACE2, the SARS-CoV-2 receptor. Notably, plant-produced Endo-H-deglycosylated RBD exhibited stronger binding to ACE2 compared with its glycosylated counterpart.

Importantly, in this study, both plant-produced glycosylated and in vivo deglycosylated RBD variants of SARS-CoV-2 induced potent neutralizing responses in mice. Although SARS-CoV-2 RBD variants have been recently produced in *N. benthamiana* by different research groups that produced their different amino acid regions, none of the groups other than Siriwattananon et al. (2021) reported on virus neutralization activity of the plant-produced RBD variants. However, because mice were immunized with different RBD antigens (two 5 μg doses in our study and two 10 μg doses in the 2021 study by Siriwattananon et al. [31]), it is not possible to compare the neutralization titers between two RBD variants.

In conclusion, all our data support that plant-produced RBD antigens are promising vaccine candidates for the prevention of COVID-19. These plant-produced antigens can also be used as a diagnostic reagent in serological tests for detection of SARS-COV-2 antibodies in COVID-19 patients.

## Data Availability

Not applicable.

## Supplementary Materials

Supplementary materials can be found at https://www.mdpi.com/article/10.3390/v13081595/s1. Figure S1: Western blot and SDS-PAGE analysis of His6-tagged RBD. (A) Western blot analysis of a crude extract of the plant *N. benthamiana* infiltrated with pEAQ-RBD-His6. (B) SDS-PAGE analysis of Ni-column purified His6-tagged RBD protein, purified from the plant *N. benthamiana*.
