# Supplementary material for: Plant-Produced Glycosylated and In Vivo Deglycosylated Receptor Binding Domain Proteins of SARS-CoV-2 Induce Potent Neutralizing Responses in Mice
Source: Viruses. 2021 Aug 12;13(8):1595. doi: 10.3390/v13081595 (PMC8402646; doi:10.3390/v13081595)
Supplement: Supplementary file 1 [file viruses-13-01595-s001.zip › viruses-1317305 suppl final.pdf]

Supplementary Materials

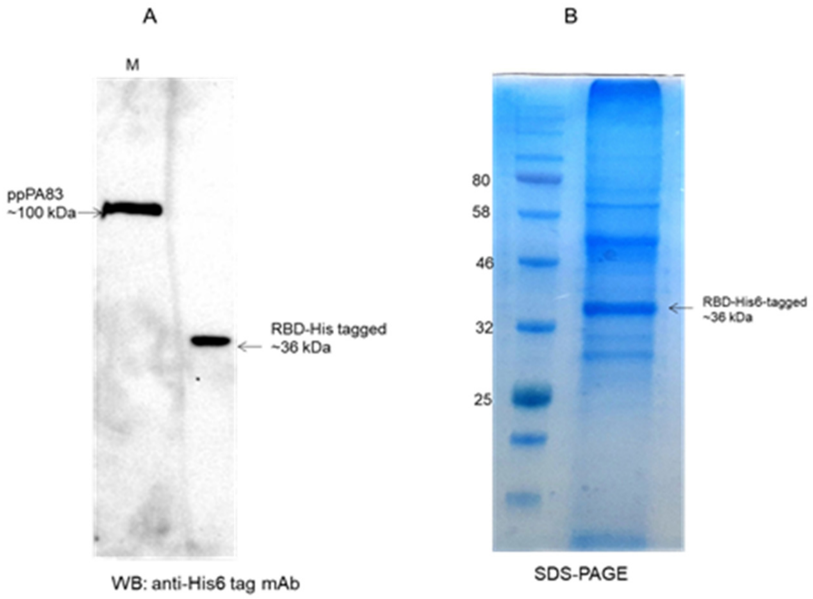

Figure S1. Western blot and SDS-PAGE analysis of His6-tagged RBD. (**A**) Western blot analysis of crude extract of *N. benthamina* plant infiltrated with pEAQ-RBD -His6. (**B**) SDS-PAGE analysis of Ni-column purified RBD-His6 tagged protein, purified from N. *benthamiana* plant.
